# Comment on: “Effect of vitamin D monotherapy on indices of sarcopenia in community‐dwelling older adults: a systematic review and meta‐analysis” by Prokopidis et al.

**DOI:** 10.1002/jcsm.13038

**Published:** 2022-07-08

**Authors:** Shih‐Hao Cheng, Chiehfeng Chen, Woei‐Chyn Chu, Yi‐No Kang

**Affiliations:** ^1^ Department of Biomedical Engineering National Yang‐Ming University Taipei Taiwan; ^2^ Department of Orthopedics Cheng Hsin General hospital Taipei Taiwan; ^3^ Cochrane Taiwan Taipei Medical University Taipei Taiwan; ^4^ Evidence‐Based Medicine Center, Wan Fang Hospital Medical University Hospital Taipei Taiwan; ^5^ Division of Plastic Surgery, Department of Surgery, Wan Fang Hospital Taipei Medical University Taipei Taiwan; ^6^ Department of Public Health, School of Medicine, College of Medicine Taipei Medical University Taipei Taiwan; ^7^ Research Center of Big Data and Meta‐analysis, Wan Fang Hospital Taipei Medical University Taipei Taiwan; ^8^ Institute of Health Policy & Management, College of Public Health National Taiwan University Taipei Taiwan

It was interesting to read a synthesis of effects of vitamin D monotherapy on sarcopenia in community‐dwelling older adults by Konstantinos Prokopidis *et al*. (2022).^1^ Doctor Prokopidis *et al*. nicely included 10 randomized controlled trials for meta‐analysis on this topic, and most findings reported indicate that vitamin D monotherapy could not improve sarcopenia outcomes in community‐dwelling older population. These findings could be understood due to mono‐use of vitamin D, and combination of vitamin D with protein intake and exercise would be a better strategy for patients with sarcopenia according to prior experience.^2^ We have noticed that the authors tried to extensively confirm the effects of vitamin D monotherapy using a composite outcome (general physical performance), and they also pooled data in an appropriate approach by standardized mean difference (SMD). However, a high heterogeneity (*I* ^2^ = 71%) may need a further discussion. According to previous evidence, age might be a potential factor due to the associations between them.^3^, ^4^, ^5^

**Figure 1:**
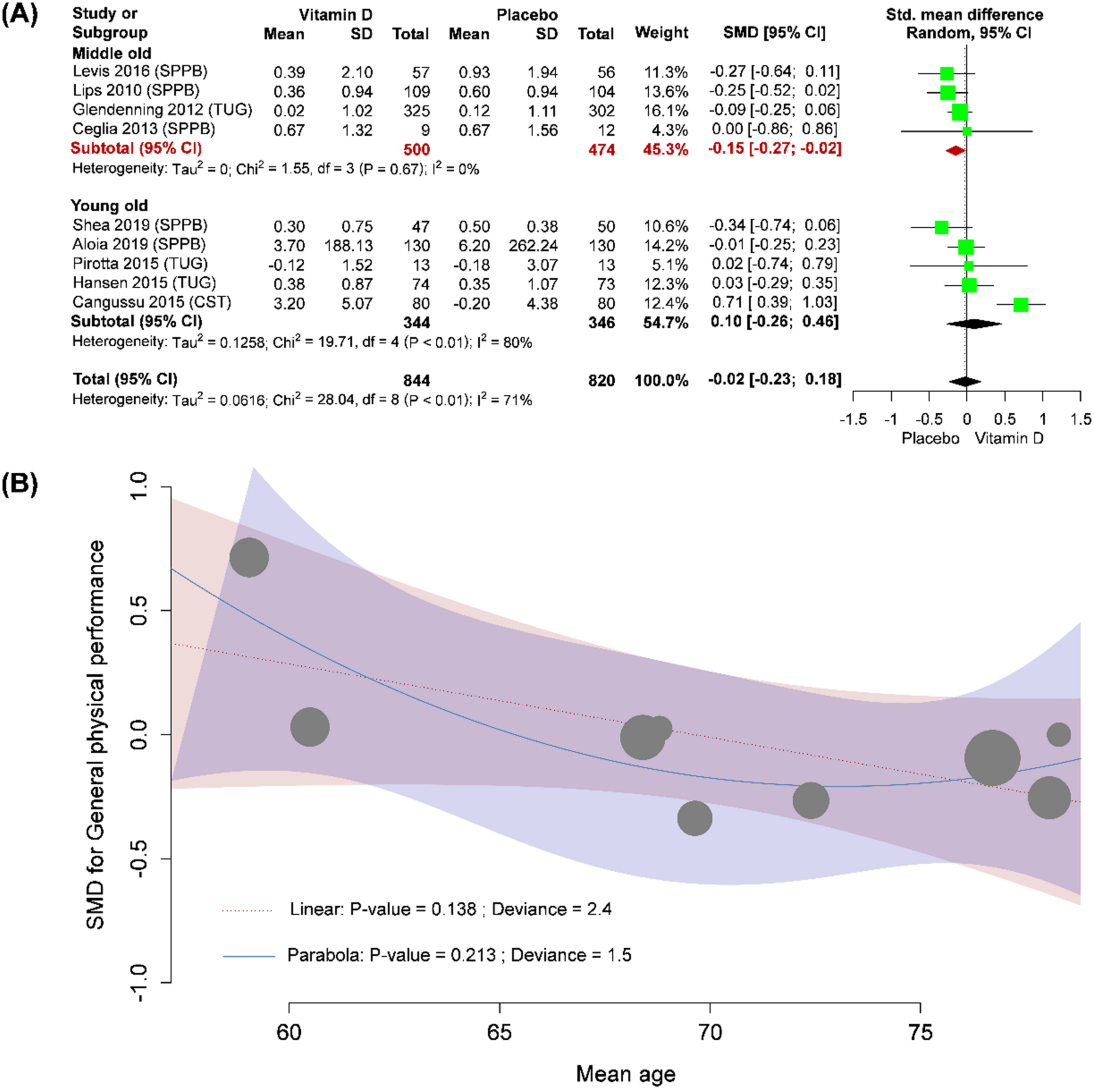
General physical performance (A) between vitamin D and placebo, and (B) association between effects of vitamin D and age. CI, confidence interval; SD, standard deviation; SMD, standardized mean difference.

We did further analysis based on the data set that were reported by Prokopidis *et al*. (2022),^1^ and analysed data in random‐effects model with subgroup by mean age. Because older population could be classified into young‐old (60 to 69 years old), middle‐old (70 to 79 years old) and old‐old (≥80 years old),^6^ this further analysis took the threshold for subgroup analysis. To depict trend between effects of vitamin D and age, we also used meta‐regression. All analysis were carried out by R version 4.1.0 via RStudio version 1.41717. In addition to the reproduced overall pooled results (SMD: −0.02; 95% CI: −0.23 to 0.18), the present analysis found reduced heterogeneity in a subset of middle‐old population (*Figure* 1A). Moreover, our subgroup analysis showed that vitamin D appeared to significantly decrease general physical performance in middle‐old population (SMD: −0.15; 95% CI: −0.27 to −0.02), but there was no significant difference between vitamin D monotherapy and placebo in subgroup of young‐old population. Result of meta‐regression also reflected the phenomenon (particularly the trend in non‐linear model due to a lower deviance), although it did not reach statistical significance (Figure 1B). These findings are similar to a previous important study.^7^

In conclusion, this further analysis fosters the understanding of the effects of vitamin D monotherapy for sarcopenia outcome in community‐dwelling older population through exploring the influence of age. Vitamin D supplement may have different effect in older population of different age. This hypothesis may explain the divergent conclusion of previous study on vitamin D in preventing falling. Nevertheless, subgroup analysis could not fully explain the heterogeneity in young‐old population. Regression would be a better method to identify the association between effects of vitamin D monotherapy and age, while our analysis has no power to find the trend due to very limited numbers of studies included in the meta‐regression. Wherefore, large‐scale studies are still needed to reveal how the effects of vitamin D on sarcopenia outcomes in community‐dwelling older population, and identify which age group should take vitamin D cautiously.
